# In Which Situations Do We Eat? A Diary Study on Eating Situations and Situational Stability

**DOI:** 10.3390/nu15183967

**Published:** 2023-09-14

**Authors:** Patricia Wowra, Tina Joanes, Wencke Gwozdz

**Affiliations:** 1 Department of Consumer Research, Communication & Food Sociology, Justus Liebig University, 35390 Giessen, Germany; tina.joanes@haushalt.uni-giessen.de (T.J.); wencke.gwozdz@fb09.uni-giessen.de (W.G.); 2 Department of Management, Society and Communication, Copenhagen Business School, 2000 Frederiksberg, Denmark

**Keywords:** situation, context, food environment, eating behavior, meal pattern, diary study

## Abstract

Eating situations are crucial for understanding and changing eating behavior. While research on individual situational dimensions exists, little is known about eating situations as a whole. This study aimed to fill this gap by identifying eating situations as combinations of multiple situational dimensions and describing how stable individuals eat in those situations. In a five-day online diary study, 230 participants reported a total of 2461 meals and described the corresponding eating situation using predefined situational dimensions. Divisive hierarchical cluster analyses were conducted separately for breakfast, lunch, and dinner, resulting in unique cluster solutions that characterized the most common eating situations. The most common breakfast situations were characterized by a combination of the dimensions social, affect, and hunger. The most common lunch and dinner situations were characterized by varying combinations of the dimensions social, affect, and activity. Based on the identified situations, a situational stability index was developed to describe how stable individuals eat in the same situations. The findings suggest high interindividual differences in situational stability, which were associated with socio-demographic characteristics like age or employment. This study enhances our understanding of the situational aspects of eating behavior while offering tools to describe eating situations and situational stability.

## 1. Introduction

Over the past decades, Western diets have fallen short in terms of quantity, quality, and diversity [1]. This shortfall is also noticeable in the context of meal consumption. Meals are defined as regular and planned eating episodes that include multiple foods and a beverage [2]. They form an integral part of Western eating behaviors, with breakfast, lunch, and dinner being traditional meal types [3]. Current trends in meal consumption include the supersizing of portions [4,5,6,7], the wide availability of fast and highly processed foods [8,9,10,11], and the decline in dietary diversity [12]. These unhealthy trends have been linked to chronic non-communicable diseases, including obesity [1,11,13,14]. To mitigate such health outcomes and promote healthy diets, understanding the determinants of meal consumption is necessary. 

Among the determinants of meal consumption, one holds particular significance as it is temporally and physically close to the behavior: the eating situation. The eating situation describes the specific circumstances in which eating occurs and is composed of situational dimensions internal (e.g., hunger or affect) and external to the person (e.g., temporal or social) [2]. Eating situations have been widely recognized as having a powerful impact on our eating behavior [2,15,16,17,18,19,20,21]. Accordingly, what we eat and why we eat vary not only between people but also between eating situations [22]. 

To better understand how eating situations impact eating behavior, it is essential to identify the situations in which people eat. To date, little is known about eating situations as a whole. Previous approaches to investigating eating situations have (1) either focused on individual situational dimensions (e.g., social—[23] or location—[24]), (2) explored multiple situational dimensions but independently from each other (e.g., location, social, and affect—[25] or time and location—[17]), or (3) preselected specific eating situations (e.g., eating out in fast-food restaurants—[26] or eating during a lunch break at work—[27]). While these approaches provided valuable insights into situational aspects of eating behaviors, they were associated with several shortcomings. First, focusing on an individual situational dimension limits understanding the multitude of situational influences on eating. Second, independently exploring multiple situational dimensions fails to account for the complex interactions between dimensions in real-life eating situations. Finally, investigating preselected eating situations may not accurately capture the diversity of eating situations individuals encounter daily. These limitations highlight the need for a more comprehensive and holistic approach to studying eating situations.

In addition to identifying eating situations, it is valuable to explore how stable these situations are for an individual. This so-called situational stability refers to the recurrence of eating situations within an individual over time. To illustrate, suppose an individual recurrently eats breakfast in the same situation (e.g., eating breakfast alone daily at 8 a.m. while working in the office). In that case, their eating behavior can be described as situationally stable. Conversely, suppose an individual eats breakfast in varying situations (e.g., sometimes at home with the family, sometimes alone in the office). In that case, their eating behavior can be described as situationally unstable. 

Investigating situational stability is important, as it complements research on the underlying processes driving eating behaviors, ranging from deliberate to automatic [28]. Previous research has associated situational stability with automatic processes across different behaviors [29,30,31]. These automatic processes are considered more resistant to change. For example, recurrently eating breakfast in the same situation might make the behavior more automatic and potentially more difficult to change [30]. In such cases, interventions could target the recurring situation to facilitate effective behavior change. However, the degree to which eating occurs in stable situations remains largely unexplored. This study aims to bridge this gap by exploring situational stability. 

Furthermore, situational stability is likely to vary between individuals depending on socio-demographic characteristics. For instance, parents with young children may prefer to adhere to a strict eating pattern, whereas students may need to be more flexible with their eating patterns depending on their schedule. Examining situational stability across socio-demographic groups facilitates the design of interventions by identifying different target groups and addressing their needs. To the best of our knowledge, there are no studies on situational stability and its relation to different socio-demographic groups.

In summary, the objectives of this study are twofold. The first research objective (RO1) is to identify the most common eating situations as combinations of multiple situational dimensions. The second research objective (RO2) is to describe situational stability and its association with socio-demographic characteristics. Each research objective will be analyzed separately for each meal type (breakfast, lunch, and dinner). 

To achieve these research objectives, (a) a multidimensional framework was applied to define eating situations; (b) a repeated-measures design was employed to capture real-life situations; and (c) a data-driven methodology was utilized to identify the most common eating situations. 

**Figure 1:**
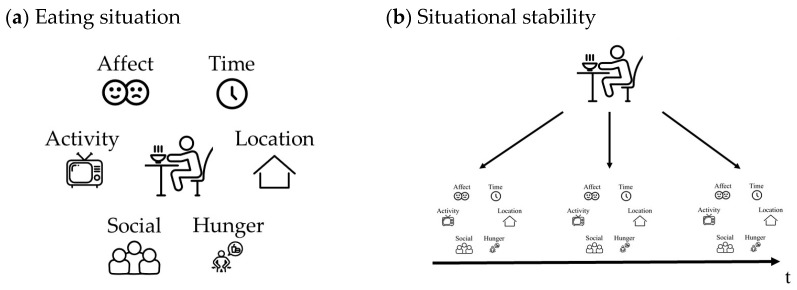
Eating situation and situational stability. The figure depicts two images. (**a**) The left image illustrates the concept of an eating situation comprising six situational dimensions, including time, location, hunger, social, activity, and affect. (**b**) The right image illustrates situational stability, which refers to the recurrence of eating situations within an individual over time. The figure has been adapted from Bisogni et al. (2007) [2].

(a) The multidimensional framework used to define eating situations was introduced by Bisogni et al. [2]. They describe eating situations as comprising seven situational dimensions: time, location, hunger, social, activity, affect, and situational stability. A depiction of the framework can be seen in Figure 1. The left image illustrates the first six situational dimensions that together describe the immediate eating situation. Each situational dimension can be further characterized by features that describe the eating situation in more detail. For example, the situational dimension of time can be described more precisely with features like “8 a.m.” or “after waking up”. The right image depicts the dimension of situational stability. Although situational stability is listed as one of the seven situational dimensions in the framework, it must be considered separately as it represents an aggregation of eating situations within an individual over time. Applying this framework addresses the limitation of examining individual situational dimensions in isolation. Instead, it characterizes eating situations as a combination of multiple situational dimensions, facilitating a holistic perspective on eating situations.

(b) Another notable feature of this study is the repeated-measures design as a diary study. In a diary study, individuals describe their eating situations repeatedly across multiple days. This design is well-suited for studying repeated-occurrence behaviors, such as eating, that recur frequently in various situations [32,33]. The diary study enables data collection close to the individuals’ experiences, providing a realistic reflection of their eating situations. Also, it enables obtaining a large sample of eating situations within individuals [32,33], thus capturing the diversity of eating situations [34]. The repeated-measures design addresses the limitations of previous research that relied on cross-sectional designs when investigating situational aspects of eating. Additionally, the diary study permits the description of situational stability, for which multiple observations across time for each individual are needed.

Last, (c) the study utilizes a data-driven methodology of cluster analyses to identify eating situations by identifying patterns of co-occurring situational dimensions. Unlike approaches that focus on situational dimensions independently or limit the study to a preselected set of situations, this methodology allows for the discovery of naturally occurring combinations of situational dimensions. By considering these combinations, the data-driven approach provides a nuanced understanding of eating situations [2].

## 2. Materials and Methods

A repeated-measures design as a diary study was employed. Data were collected by the German market research company Aproxima on weekdays from the 11th until the 15th of January 2021. A five-day time frame was decided upon to capture a whole working week. Participants had access to the online questionnaire each day from 7 p.m. until noon the following day and were instructed to fill out the questionnaire after their last meal of the day. On the first day, participants were informed about the theme of the study (investigating eating behavior) and received instructions for the diary study. They were also asked for consent to participate in the study and completed a short intake survey on socio-demographic characteristics (age, sex, employment, household composition, and income).

### 2.1. Participants

The participants were eligible to take part in the study if they were at least 18 years old and did not follow a vegetarian or vegan diet. All participants were compensated for each day they took part in the diary study. Compensation was staggered so that more money was offered on the first and last days to motivate participants to complete the study (in total, EUR 3.80). Of the 481 participants starting the study on the first day, 230 completed all five days (response = 47.82%). Drop-out analysis found socio-demographic differences between participants who completed the five days and those who dropped out; see Table S1 in the Supplementary Material. Participants who worked part-time or did not work were more likely to complete the study than those who worked full-time. Also, participants who lived in households without children were more likely to complete the study than those who lived with children. Only the 230 participants who completed the diary entries for all five days were included in the analyses. The socio-demographic characteristics of the final sample are presented in Table 1.

### 2.2. Measures

In the daily diary survey, participants reported how many meals they had eaten that day (excluding snacks), assigned each to a meal type (breakfast, lunch, dinner), and described the situation in which they had eaten their meal. Participants characterized the situations using the six situational dimensions (time, location, hunger, social, activity, and affect) of the multidimensional framework, which was outlined by Bisogni et al. [2]. Each situational dimension was assessed with one item to ensure a short response time for the participants. Only the dimension affect was assessed with a short scale, which was subsequently transformed into a single numerical value. Furthermore, all situational dimensions with numerical values were dichotomized. These steps were implemented to give each situational dimension equal weight in the analyses. 

The time of day was measured by a drop-down menu in hourly intervals. Descriptive analysis revealed a trimodal distribution, meaning that eating occurred during three peak times: morning, midday, and early evening. These peaks were significantly associated with the self-reported meal types (breakfast, lunch, and dinner), *X*^2^(4, *N* = 2461) = 4007.3, *p* < 0.001. Thus, time was dropped as a situational dimension.

The location was assessed with the question about where the meal was taken with the following options: “at home”, “at someone else’s home”, “in a canteen, cafeteria, fast-food chain, restaurant, a snack bar or similar”, “at the workplace”, “on the road (e.g., on the bus, in the train, in the car, …)”, “outside in nature (e.g., a park)” or “in another place”. Due to low counts in all other locations apart from “at home”, they were combined into a single category, referred to as “elsewhere”. The low counts of options outside the home can potentially be explained by the impact of the COVID-19 pandemic and the associated restrictions on eating behavior. Consequently, the final options for location were “home” and “elsewhere”. 

Hunger prior to meal consumption was measured by a unipolar visual analog scale, from 0 (not at all hungry) to 100 (extremely hungry) [35]. Hunger was dichotomized into “hungry” and “satiated” for each meal type using a median split. The median score across all meals was 62. Specifically, the median score for breakfast was 60, for lunch it was 65, and for dinner it was 64. 

The social dimension was measured by whether other people had been present while eating. Participants could answer either “with others” or “alone”.

For activity, participants indicated whether they did something else in addition to eating and could answer either “with activity” or “without activity”. The participants were given examples of activities such as watching TV, browsing the Internet, or working. 

The affect was assessed by a shortened version of the positive and negative affect schedule (PANAS; [36,37]). The positive affect (PA) subscale consisted of the three positive affect items: happy, relaxed, and energized. The negative affect (NA) subscale consisted of the three negative affect items: angry, afraid, and sad. Participants rated how they felt on a 7-point scale, ranging from 1 (not at all) over 4 (moderately) to 7 (extremely). Both subscales (PA and NA) emerged as separate factors tested by multilevel factor analysis: *X*^2^(16) = 113.4; CFI = 0.96; TLI = 0.93; RMSEA = 0.05. To avoid prioritizing the situational dimension of affect in the analyses by including both PA and NA, only NA was kept as a proxy for affect. This decision was supported by literature demonstrating the importance of negative affect for eating behavior [38,39,40]. For the analysis, the affect was dichotomized into “high negative affect” and “low negative affect” separately for each meal type using a median split (the median was 1 across all meals and meal types). 

### 2.3. Statistical Analysis

All statistical analyses were performed using R Studio (version 4.0.2). To investigate in which situations people eat their meals (RO1), a data-driven methodology was utilized by conducting divisive hierarchical cluster analyses. The cluster analyses were performed separately for each meal type (breakfast, lunch, and dinner). Within the cluster analyses, the Gower coefficient was used to measure the distance in the clustering process [41,42]. The optimal number of clusters was determined using the elbow method and the average silhouette width [42], whereby both methods always led to the same results. The resulting clusters represent the most common situations for each meal type. 

To describe situational stability (RO2), an index was developed accounting for how often an individual ate a meal in the same situation (stability) and how certainly a person can be categorized as eating in recurrent eating situations (certainty). The situational stability index ranges from 0 (very unstable) to 1 (very stable) and was calculated by the following:

$$Situational\;Stability\;Index=1-\frac{\left(N_{situations}-0.5\right)}{N_{meals}}$$

On the one hand, the index reflects how stable individuals eat in the same situation. The index has a reference value set at 0.5, which can be considered a neutral value; it indicates neither high nor low situational stability. If a person tends to eat in the same situation, the index results in values above 0.5 (high situational stability). However, if a person tends to eat in different situations, the stability index results in values below 0.5 (low situational stability). Consider the following example: Participants A and B ate five breakfasts during the diary study (*N*_meals_ = 5). Participant A always ate breakfast in the same situation (*N*_stiuations_ = 1), while Participant B ate breakfast in three different situations (*N*_situations_ = 3). Participant A would receive an index score of 0.90, while Participant B would receive an index score of 0.30. Hence, individuals score higher on the index if they eat meals in the same situation. Individuals score lower on the index if they eat meals in different situations. 

On the other hand, the index also reflects the certainty with which a person can be categorized as situationally stable or unstable in their eating patterns. Consider the comparison between Participant A, who ate five breakfasts during the diary study (*N*_meals_ = 5), always in the same situation (*N*_situations_ = 1), and Participant C, who ate breakfast twice during the five days (*N*_meals_ = 2), also always in the same situation (*N*_situations_ = 1). Although both participants ate stably in the same situation, they differed in the number of reported meals. Participant A’s eating behavior is well documented (featuring five breakfasts), and it can be inferred with high certainty that this person has a high level of situational stability. In contrast, the data on Participant C’s eating behavior are limited (featuring two breakfasts). Although it seems that Participant C also eats stably in the same situation, this assumption cannot be made with the same certainty as for Participant A. Thus, the more information available about a person’s eating pattern (more meals), the more certain one can infer whether that person tends to eat in the same or different situations. The index reflects this difference in certainty by correcting the number of reported meals. In this example, Participant A would receive a higher situational stability score (stability index = 0.90) than Participant C (stability index = 0.75). 

In terms of certainty, the case of one meal (*N*_meals_ = 1) is interesting. If a person eats breakfast only once during the observed period, no assumptions can be made about whether this person eats breakfast in the same or different situations. There were not enough data to categorize their situational stability. Due to the uncertainty in categorizing participants who report only one meal, the index will result in a score of 0.5. 

The index is calculated separately for each meal type (breakfast, lunch, and dinner) to account for varying eating patterns depending on the meal type. For example, a person might always eat breakfast in the same situation (alone at home) but dinner in different situations (sometimes alone and sometimes with friends). The situational stability index as a function of meals and situations is exemplified in Table 2.

In addition, the association between the situational stability index and socio-demographic characteristics was examined (RO2). For this purpose, correlation analyses (Spearman’s rank correlations) and mean comparison tests (Mann–Whitney U tests and Kruskal–Wallis tests) were conducted. Non-parametric tests were chosen due to the non-normal distribution of the situational stability index. Subsequently, pairwise comparisons were performed using post hoc Dunn tests with Bonferroni correction.

## 3. Results

On average, participants reported 10.74 meals over 5 days, including about 3.35 breakfasts, 3.45 lunches, and 3.9 dinners; see Table 3. Most meals were eaten at home, with dinner being the most frequent and lunch the least. Regarding the other situational dimensions—hunger, social, activity, and affect—no clear pattern emerged, as about half of the meals were eaten in either of the two options. For example, about half of the meals were eaten hungry and the other half satiated. These findings were consistent across all meals, although variations in social and activity were observed between the meal types. More dinners were eaten with others than lunches, and slightly more lunches were eaten with others than breakfasts. Also, more breakfasts and dinners were accompanied by an activity than lunches. For a detailed description of the situational dimensions individually and across all meals, see Table 4.

### 3.1. RO1: Eating Situations for Breakfast, Lunch, and Dinner

The cluster analyses were conducted to identify the most common eating situations. Eating situations can be described by varying combinations of situational dimensions (location, hunger, social, activity, and affect). In the following section, the results of the cluster analyses will be presented separately for breakfast, lunch, and dinner. For each meal type, the most common eating situations will be briefly described (N > 100 meals/situation). Descriptive data on all eating situations for each meal type can be found in the Supplementary Material (see Tables S2–S4).

#### 3.1.1. Breakfast

**Figure 2:**
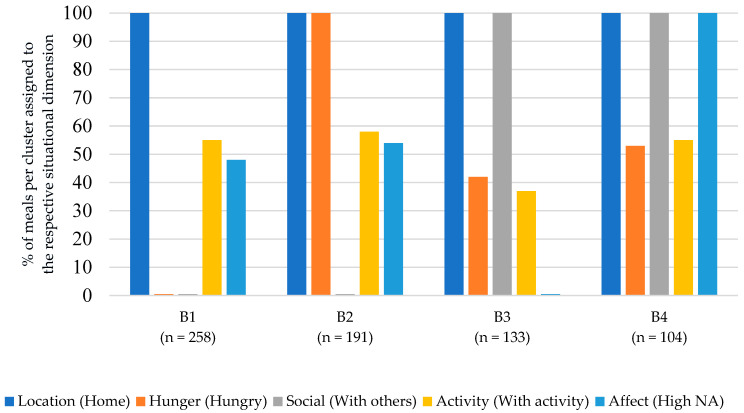
Description of the four most common eating situations for breakfast.

The cluster analysis for breakfast yielded a six-cluster solution (see Figure 2). The most common situations were labeled according to their dimensions: (B1) Breakfast at home, satiated and alone (34% of all breakfasts); (B2) Breakfast at home, hungry and alone (25%); (B3) Breakfast at home, with others and a low negative affect (17%); (B4) Breakfast at home, with others and a high negative affect (14%). 

Situations B1 and B2 described situations in which breakfast was eaten at home and alone. The main difference between the two situations resulted from the situational dimension of hunger: B1 described situations in which breakfast was eaten satiated, while B2 described situations in which breakfast was eaten hungry. The dimensions of activity and affect played a minor role in defining these breakfast situations.

Situations B3 and B4 described situations in which breakfast was eaten at home and with others. The situations differed in affect: B3 was characterized by a low negative affect, whereas B4 was characterized by a high negative affect. The dimensions of hunger and activity played a minor role in defining these breakfast situations.

#### 3.1.2. Lunch

**Figure 3:**
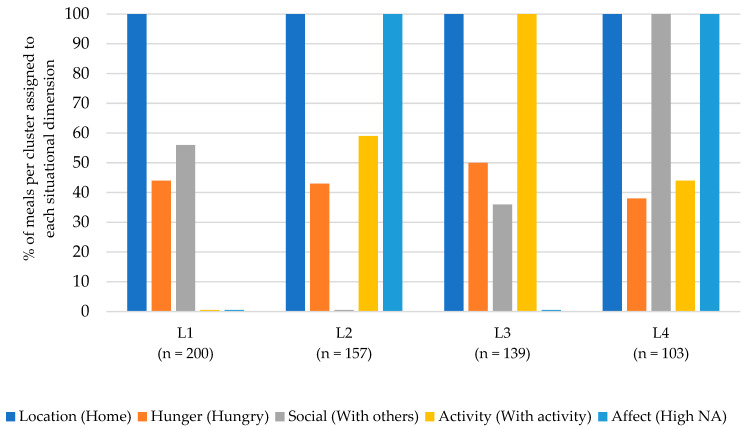
Description of the four most common eating situations for lunch.

The cluster analysis for lunch resulted in an eight-cluster solution (see Figure 3). The most common situations were labeled according to their dimensions: (L1) Lunch at home, no other activity, and a low negative affect (25% of all lunches); (L2) Lunch at home, alone, and a high negative affect (20%); (L3) Lunch at home with another activity and a low negative affect (18%); (L4) Lunch at home with others and a high negative affect (13%). 

Situations L1 and L3 both described situations in which lunch took place at home with a low negative affect. The situations differed in terms of activity: L1 described situations in which eating was the sole activity, while L3 described situations in which meals were accompanied by another activity. Also, the situations differed slightly regarding the social dimension: In L1, a slightly higher portion of lunches were eaten with others compared to L3. The dimension of hunger played a minor role in defining these lunch situations.

Situations L2 and L4 described situations in which lunch was eaten at home with high negative affect. The situations differed in terms of the social dimensions: L1 described situations in which lunch was eaten alone, while L4 described situations in which lunch was eaten with others. The dimensions of hunger and activity played a minor role in defining these situations.

#### 3.1.3. Dinner

**Figure 4:**
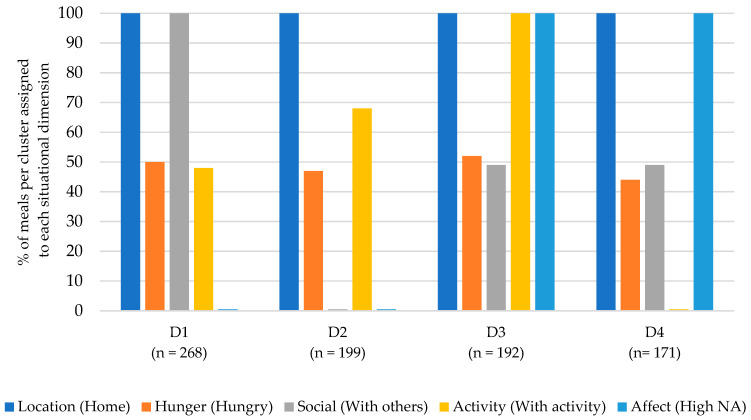
Description of the four most common eating situations for dinner.

The cluster analysis for dinner resulted in a seven-cluster solution (see Figure 4). The most common situations were labeled according to their dimensions: (D1) Dinner at home with others and a low negative affect (30% of all dinners); (D2) Dinner at home, alone and a low negative affect (22%); (D3) Dinner at home, with another activity and a high negative affect (21%); (D4) Dinner at home with no other activity and a high negative affect (19%). 

Situations D1 and D2 described situations in which participants ate dinner at home with low negative affect. The situations differed in terms of the social dimension: D1 described situations in which dinner was eaten with others, while D2 described situations in which dinner was eaten alone. Also, the situations differed slightly in whether dinners were accompanied by another activity (D2) or not (D1). The dimension of hunger played a minor role in defining these situations.

Situations D3 and D4 described situations in which dinner was eaten at home with a high negative affect but differed in activity: D3 described situations in which dinner was accompanied by another activity, while D4 described situations in which eating dinner was the sole activity. The dimensions of hunger and social played a minor role in defining these situations.

### 3.2. RO2: Situational Stability and Its Association with Socio-Demographic Characteristics

Next, situational stability and its association with socio-demographic characteristics were explored. The situational stability index was calculated per person based on the previously identified situations. In this study, the mean situational stability index was above the reference value of 0.5 for each meal type, indicating that the sample tended to eat in recurring situations for breakfast, lunch, and dinner (see Table 5). As the mean stability index was similar across all meal types (between 0.62 and 0.68), it can be concluded that there were no differences in situational stability between breakfast, lunch, and dinner. Also, the degree of situational stability differed considerably among participants, with some having unstable eating patterns (e.g., dinner: 0.10) and others having very stable eating patterns (e.g., dinner: 0.93). This indicates high interindividual differences in situational stability.

Regarding the association between situational stability and socio-demographic characteristics, the results suggest that situational stability was associated with age, employment status, and adults and children in the household (see Table 6). Age was positively correlated with situational stability across all meal types, indicating that the older the participants, the more stably they ate their meals in the same situation. The association between employment status and situational stability varied depending on the meal type. For breakfast, participants in education had lower situational stability than those employed full-time (*p* < 0.05) or those not working (*p* < 0.001). For lunch, participants who were not working had higher situational stability than those employed full-time (*p* < 0.05). However, for dinner, no association between employment status and situational stability was found. Adults and children in the household were also associated with situational stability, but only at breakfast. Although the overall test showed an association between adults in the household and situational stability, none of the pairwise comparisons in the post-hoc tests were significant after controlling for multiple tests. Participants living without children scored higher on situational stability during breakfasts than those living with children (*p* < 0.05). Finally, participants’ sex and income were unrelated to situational stability, regardless of the meal type.

## 4. Discussion

The present study offers novel insights into the situational aspects of meal consumption. By adopting the multidimensional framework proposed by Bisogni et al. [2], multiple situational dimensions were considered and combined to compose eating situations. By employing a repeated-measures design and a data-driven methodology, the variety of daily eating situations was captured and the most common ones identified. Furthermore, the stability of these eating situations was explored by developing a situational stability index. The association between situational stability and social-demographic characteristics, such as age or employment, was investigated. In the following, the implications of these findings and directions for future research will be discussed. 

### 4.1. Eating Situations as Combinations of Situational Dimensions

The first objective of this study was to identify eating situations. Eating situations were characterized by unique combinations of situational dimensions. The uniqueness of each eating situation was determined by the specific combinations of different situational dimensions and their respective features, which varied depending on the meal type. This approach provided a more detailed picture of eating situations compared to describing individual situational dimensions independently. In this section, the study’s findings will be revisited in the context of previous research, and the added insights provided by this approach will be illustrated.

#### 4.1.1. Breakfast

This study found that the two most common breakfast clusters (B1 and B2) were characterized by eating at home and alone. This finding is consistent with previous research on individual situational dimensions, indicating that most breakfasts are eaten at home and primarily alone [43,44,45,46,47]. Moreover, the finding extends existing research by highlighting the combinations of situational dimensions, specifically between social and either hunger (for B1 and B2) or affect (for B3 and B4), in defining the most common breakfast situations. 

Despite previous literature suggesting that individuals often engage in other activities while eating breakfast [48], this study’s findings did not indicate that activity played a defining role in characterizing the four most common breakfast situations. Instead, activity in combination with social and affect served as the basis for defining the two minor breakfast situations (B5 and B6); see Table S2 in the Supplementary Material. 

#### 4.1.2. Lunch

Previous research on lunch situations has consistently emphasized the role of the dimensions social and activity [18,49]. Studies focusing on individual situational dimensions have observed that about half of all lunches are eaten when other people are present, and most lunches are eaten while engaged in another activity [47,48,50]. Also, prior research has identified lunch situations characterized by a combination of the dimensions social and activity, such as eating with family while watching TV [18,49]. The findings align with previous studies while also expanding upon them. Specifically, they highlight the role of social and activity in combination with affect when defining lunch situations (see L1–L4). 

Interestingly, the findings emphasize the combination of activity and affect in defining lunch situations. This combination was observed for meals eaten at home (L1 and L3: both with low negative affect but differed in activity) and outside the home (L7 and L8: both accompanied by activity but differed in negative affect) (see Table S3 in the Supplementary Material). Taken together, nearly 50% of the observed lunches involved a combination of activity and affect, underscoring their importance in investigating lunch situations.

#### 4.1.3. Dinner

Similar to lunch, previous research on dinner situations has also highlighted the combination of the dimensions social and activity [18,49]. For children and adolescents, common eating situations include eating a family meal while watching TV, eating while watching TV, or eating while being with friends. The present study is consistent with previous research regarding the importance of social and activity, but variations in the specific combinations of these situational dimensions were observed. This discrepancy could be attributed to various factors, including differences in samples (adults vs. children and adolescents) or differences in situational dimensions (e.g., the present study included affect as a situational dimension, which, in combination with social and activity, made up the most common eating situations). These differences in findings across studies warrant more research on dinner situations using a comprehensive set of situational dimensions.

#### 4.1.4. Summary

In summary, this study found unique combinations of situational dimensions that made up the most common eating situations. For breakfast, combinations of hunger, social, and affect resulted in the most common eating situations, while for lunch and dinner, varying combinations of social, activity, and affect defined the most common eating situations. Two aspects stand out: First, the combination of social and affect played a significant role in defining eating situations across all meal types. The most common eating situations unique to each meal type were then distinguished by combining social and affect with other situational dimensions. For breakfast, the most common eating situations were characterized by combinations of social, affect and hunger. For lunch and dinner, the most common eating situations were characterized by combinations of social, affect, and activity. 

Second, although the eating situations for lunch and dinner shared the same set of situational dimensions, they differed in the specific combinations of these dimensions. For lunch, eating situations with high negative affect differed depending on whether individuals ate alone or with others, while eating situations with low negative affect differed depending on whether individuals engaged in another activity while eating. For dinner, the pattern was reversed: eating situations featuring high negative affect differed depending on whether individuals engaged in another activity while eating, and situations with low negative affect differed depending on whether individuals ate alone or with others. These differences in combinations might result from distinct features prevalent during lunch and dinner. For example, for social, lunch might be eaten with co-workers, while dinner might be eaten with family. Further investigation is needed to fully understand these differences. 

Overall, examining combinations of situational dimensions can be a valuable addition to the mere description of individual situational dimensions. By identifying patterns of co-occurring situational dimensions, real-life eating situations can be more accurately represented. This enables researchers and practitioners to better understand the situations in which people eat and how different situational dimensions are interrelated. 

Identifying eating situations can benefit behavior change interventions promoting healthy eating. By pinpointing the specific situations in which individuals eat, researchers and practitioners can prioritize their resources to address the most common eating situations. This approach acknowledges that eating behaviors are situation-dependent and that interventions need to be tailored to the unique situation in which they occur. In order to tailor interventions to situations, researchers should decide on a specific meal type. This is supported by the findings of this study and previous research, which have highlighted that eating situations vary greatly depending on the meal type [18,49]. Interventions should, therefore, focus on the most common situations per meal type. For example, for breakfast, researchers may target eating at home alone while feeling satiated (B1). Tailoring interventions to the most common eating situations associated with each meal type could enhance their effectiveness in changing behavior. 

### 4.2. Stability of Eating Situations within and between Individuals

The second objective of this study was to describe situational stability and explore its association with socio-demographic characteristics. For that, a situational stability index was developed to examine how often individuals eat in the same eating situations. The findings demonstrate considerable variation in situational stability between individuals, indicating that some people tended to eat in the same situations while others tended to eat in varying situations. 

The analysis further revealed different socio-demographic characteristics associated with situational stability, including age, employment status, and adults and children in the household. Regarding age, this study found that the older the participants, the more stable their eating situations were, regardless of meal type. Older adults may have developed more homogeneous daily routines and adhered to them more strictly than younger adults [51]. In contrast, younger adults may have more varied daily routines (e.g., irregular schedules during their education) and may strive for flexibility, including in their eating routines (e.g., eating out with friends at new places). Regarding employment status and adults and children in the household, this study found different associations depending on the meal type. These differences may be attributed to differences in timing, demands related to work, school, or unemployment, or other people’s needs that affect the establishment and maintenance of eating routines. For example, parents may need to be more flexible in the mornings to accommodate the children’s needs, leading to less situational stability during breakfast. These findings highlight the role of socio-demographic characteristics in shaping the recurrence of eating situations.

In addition to the findings presented in this study, the stability index can be of use for future research. The following sections discuss the interpretation of the index, its contribution to research on processes determining eating behavior, and some conceptual considerations about the stability index. 

Regarding the interpretation of the index, it is essential to clarify that situational stability does not indicate the quality of one’s diet. Eating in recurring eating situations should not be automatically equated with a healthy diet, nor should eating in varying situations be equated with an unhealthy one. Situational stability merely indicates how often individuals eat in the same situations. Future research will have to evaluate the impact of situational stability on eating behaviors and health outcomes. 

That said, situational stability can complement research on the processes determining eating behavior, which encompass a spectrum from automatic to deliberative [28]. These processes might be linked to different levels of situational stability [29]. On the automatic end of the spectrum, eating behavior might be structured by fixed daily routines or automatically triggered by habits. For example, people might eat dinner because they adhere to the family’s eating schedule (daily routines) or because they always eat dinner at 8 p.m. (habits). Such automatic eating processes are often facilitated or triggered by recurring eating situations [29,30]. On the deliberative end of the spectrum, people might eat because they consciously decide to and want to satisfy varying motives, such as hunger, appetite, time constraints, social influences, and more. In this case, eating motives and the resulting eating behavior may vary greatly between different eating situations [52]. Hence, an avenue for future research could be to explore how situational stability conceptually and empirically relates to automatic or deliberate eating.

The situational stability index is a useful tool for researching situational influences on eating. The application of the index is not limited to eating behaviors during meal consumption. It can encompass other eating behaviors (such as snacking) and other populations (such as non-German samples). To do so, some adjustments to the index might be necessary. In the following, some conceptual considerations will be discussed when using or adapting the situational stability index, namely meal skipping, the optimal number of days or eating occasions, and the sampling of situations. 

An intriguing challenge for the situational stability index involves how to handle meal skipping, which refers to the omission of one or more meals throughout the day [53]. For example, Person A might only eat three breakfasts across a five-day observation period, while Person B might eat five breakfasts across the same period. Researchers need to decide whether to penalize the score for individuals who skip meals. In this study, the decision was made not to penalize for meal skipping. This decision was based on the uncertainty about the reasons for skipping meals. People may regularly skip a meal on a particular day because of their work schedule or may not even adhere to the traditional 3-meals-a-day pattern. Therefore, instead of penalizing meal skipping, the stability index was calculated as a function of the total number of meals a person reported. This approach accounts for the number of meals skipped. However, it might not be the perfect solution, and future research may need to develop more nuanced methods for incorporating meal skipping into the stability index.

A second consideration for researchers applying the situational stability index is to determine the appropriate number of occasions to observe. While this study took an exploratory approach to determining situational stability and cannot provide a specific recommendation, two suggestions can be offered when deciding on the number of occasions. First, the frequency of the behavior in question should be taken into account. If the behavior occurs often during the day, fewer days may be needed, as more observations can be made in one day. Second, the level of accuracy of the stability index required for the purpose should be considered. If the aim is to detect slight variations in situational stability, a more precise index is needed. This may require a longer period of observation. In summary, the chosen number of occasions observed should align with the research objective. 

The last conceptual note pertains to sampling situations when assessing situational stability. Just as sampling participants should strive to represent the population or a particular subgroup for valid generalizations, selecting eating situations should adhere to the same principle [54]. For that, it is important to acknowledge that eating situations within weekdays, weekends, or seasons may be more similar than those between weekdays and weekends or across different seasons [3,55]. This variation in similarity is also mirrored in eating behaviors, which differ between weekdays and weekends as well as between seasons [56,57,58,59,60]. Hence, in cases where researchers focus on a specific type of eating situation (e.g., lunches during weekdays), the stability index might skew towards higher values. However, in cases where researchers seek a broader view across different situations, the stability index may display greater variability. Therefore, the sampling of eating situations must be determined based on the researcher’s goal. 

Situational stability is also valuable when designing behavior change interventions to promote healthy eating behaviors. It offers valuable insights into which type of intervention might be most effective for which target group. Generally, interventions can target either the situation or the person. Interventions targeting the situation might be more effective for individuals with high situational stability, as eating situations are easy to target due to their stability, and the behavior might be closely linked to these situations. Such interventions could focus on modifying the recurring situations to disrupt unhealthy eating habits or pairing new eating behaviors with these situations. However, this type of intervention might be less suitable for individuals with low situational stability, as they eat in varying situations, which might be more challenging to target. In these cases, interventions could focus on the person instead of the situation. This can be carried out by addressing the motives that drive eating behavior or suggesting strategies to improve self-control or goal adherence. Here, the association between situational stability and socio-demographic characteristics might help identify homogenous groups that can benefit from the same type of intervention. Tailoring interventions to address the needs of different populations can enhance their effectiveness in promoting healthy eating behaviors.

In summary, situational stability furthers understanding the temporal dynamic of eating situations and can inform behavior change interventions. By recognizing participants with high or low situational stability, researchers and practitioners can tailor interventions to either modify the situation or support the individual to promote healthy eating behavior.

### 4.3. Strengths and Limitations

The study has three major strengths. First, it expands on previous research by not only describing individual situational dimensions but also by representing eating situations as combinations of situational dimensions that vary across meal types. 

Second, it equips future research with tools to identify eating situations and describe situational stability. These tools can be applied to other eating behaviors in addition to meal consumption (e.g., snacking) and adapted for culturally or demographically different populations (e.g., non-Germans or only employed people). 

Third, the diary study as a form of repeated measure design offers advantages in terms of methodology and content. Methodologically, obtaining multiple measurements improves data quality as participants report closer to the time their behavior occurred. This is advantageous compared to cross-sectional research, which relies on participants recalling past eating situations. Such retrospective recall can introduce recall biases due to the temporal and spatial distance from the actual eating occasion. From a content perspective, the diary method allows for analyzing situational stability as a function of multiple meals within each person. This approach offers a more accurate indicator than self-reports in cross-sectional research, where participants estimate how often they think they eat in the same situations. 

While this study offers more profound insights into eating behavior, three limitations must be addressed. First, data were collected during the COVID-19 pandemic. The pandemic disrupted eating routines on an unprecedented scale. External constraints reduced the large variety of eating situations to a minimum: homes were the central eating location, as many people worked from home and restaurants were closed; contact restrictions prevented social dinners with friends and family; and home-schooling children while working created a double burden for families. Consequently, the results of this study must be interpreted and generalized with caution. Future research should collect up-to-date data on eating situations to validate current findings and avoid restricting interpretations of eating situations to the COVID-19 period. Collecting up-to-date data will also capture potential changes in eating situations that might have occurred since the midst of the pandemic. 

The second limitation concerns the generalizability of the results, which is constrained by several factors. First, the sample consisted of German participants, and eating situations might differ across different cultural backgrounds. Second, the sample does not fully represent the entire population of Germany. Individuals from other socio-demographic backgrounds might eat in situations not adequately represented in the study. Finally, the analysis indicated differences in drop-out rates depending on socio-demographic characteristics (see Table S1 in the Supplementary Material). Different drop-out rates might introduce a bias into the data. These limitations reiterate the importance of exercising caution when generalizing the findings. Moreover, they highlight the need for more research on eating situations involving diverse samples.

The final limitation concerns the conceptualization of the situational dimensions. In this study, each situational dimension was conceptualized using only one feature. For instance, activity was defined by whether individuals engaged in an activity while eating. However, it is important to recognize that each situational dimension can encompass multiple features [2]. For instance, the dimension activity could have also been conceptualized by considering the specific type of activity, such as “watching TV” or “working”. Including additional features for each situational dimension could lead to a more precise description of eating situations. In this study, eating situations for lunch and dinner were defined by varying combinations of social, activity, and affect. However, the type of activity might vary depending on the meal type [61]. For instance, during lunch, activity might entail working while eating, whereas during dinner, it might entail watching TV. Future research could explore multiple features to provide a more detailed picture of eating situations. 

## 5. Conclusions

In conclusion, this study has contributed to understanding the situational aspect of meal consumption by shedding light on eating situations and situational stability. The findings emphasize the importance of considering combinations of situational dimensions to describe eating situations instead of exploring them in isolation. Identifying the most common eating situations for each meal type offers a realistic depiction of real-life eating situations. These insights can be used to tailor interventions that effectively address these specific situations. Furthermore, the situational stability index offers valuable insights into the variability of eating situations and can inform research on eating processes that may range from deliberative to automatic. 

The essential question of what type of food people eat in different eating situations was left unanswered in this study. Future research can expand on this study by assessing which eating situations relate to meal quantity, quality, diversity, and subsequent health outcomes. In what eating situations do we eat highly processed foods? Are there intra- and interindividual differences in situational stability for those behaviors? With the methods presented here, future research will be able to investigate the situational aspects of eating behavior in depth and thus contribute to the challenges in the field of health and sustainability. 

## Figures and Tables

**Table 1:** Socio-demographic characteristics of the final sample.

Socio-Demographic Characteristics	Mean/N	(SD/%)
Age	42.70	17.25
Sex		
Male	135	58.70%
Female	95	41.30%
Employment status		
Full-time	102	44.35%
Part-time	36	15.65%
In education	24	10.43%
Non-working	67	29.13%
Missing	1	0.43%
Household composition		
Other adults in the household		
No	37	16.09%
Yes	177	76.96%
Missing	16	6.96%
Children in the household		
No	147	63.91%
Yes	47	20.43%
Missing	36	15.65%
Monthly household net-income		
≤EUR 450	5	2.17%
EUR 450–<1500	42	18.26%
EUR 1500–<2500	64	27.83%
EUR 2500–<4000	71	30.87%
≥EUR 4000	44	19.13%
Missing	4	1.74%

**Table 2:** Values of the situational stability index as a function of meals and situations in which the meals were eaten.

Situational Stability Index		*N*_meals_^1^						
		1	2	3	4	5	6	etc.
*N*_situations_^2^	1	0.50	0.75	0.83	0.88	0.90	0.92	…
	2		0.25	0.50	0.63	0.70	0.75	…
	3			0.16	0.35	0.50	0.58	…
	4				0.13	0.30	0.42	…
	5					0.10	0.25	…
	6						0.08	…
	etc.							…

^1^ *N*_meals_ = Number of meals a person ate during the observed period. ^2^ *N*_situations_ = Number of different eating situations into which the meals were categorized.

**Table 3:** Descriptive statistics on the average number of reported meals across all five days.

	Mean	Median	SD	Range
All	10.74	11	3.56	1–22
Breakfast	3.35	4	2.10	0–11
Lunch	3.45	4	1.88	0–7
Dinner	3.9	5	1.72	0–8

**Table 4:** Frequencies and percentages of the situational dimensions overall and per meal type.

N (%)		Meal Type			
		All	Breakfast	Lunch	Dinner
	Total number of meals	2461 (100%)	770 (31.29%)	794 (32.26%)	897 (36.45%)
Situational Dimensions	Location Home (vs. elsewhere)	2115 (85.94%)	686 (89.09%)	599 (75.44%)	830 (92.53%)
	Hunger ^1^ Hungry (vs. satiated)	1220 (49.57%)	349 (45.32%)	375 (47.23%)	444 (49.50%)
	Social With others (vs. alone)	1128 (45.84%)	274 (35.58%)	364 (45.84%)	490 (54.63%)
	Activity With (vs. without activity)	1237 (50.26%)	403 (52.34%)	344 (43.32%)	490 (54.63%)
	Affect ^1^ High (vs. low negative affect)	1142 (46.40%)	337 (48.96%)	364 (45.84%)	401 (44.70%)

^1^ The situational dimensions of hunger and affect were dichotomized using a median split.

**Table 5:** Situational stability averaged per meal type.

Meal Type	N_participants_^1^	Mean	SD	Range
Breakfast	191	0.67	0.18	0.13–0.90
Lunch	200	0.62	0.20	0.13–0.90
Dinner	210	0.68	0.19	0.10–0.93

^1^ *N*_participants_ = Number of participants who reported at least one meal for the respective meal type.

**Table 6:** Association between situational stability and socio-demographic characteristics per meal type.

Socio-Demographic Characteristics	Meal Type		
	Breakfast	Lunch	Dinner
	Test Statistic ^1^ N, Mean (SD)	Test Statistic ^1^ N, Mean (SD)	Test Statistic ^1^ N, Mean (SD)
Age	*r*(189) = 0.35 ***	*r*(198) = 0.14 *	*r*(208) = 0.20 **
Sex	*U* = 4735.5	*U* = 4709.5	*U* = 5544.5
Male	112, 0.66 (0.19)	122, 0.62 (0.21)	120, 0.68 (0.20)
Female	79, 0.68 (0.18)	78, 0.63 (0.18)	90, 0.69 (0.19)
Employment status	*H*(3) = 20.28 ***	*H*(3) = 8.83 *	*H*(3) = 7.59
Full-time	83, 0.67 (0.19)	91, 0.60 (0.19)	95, 0.66 (0.19)
Part-time	28, 0.64 (0.18)	30, 0.59 (0.21)	31, 0.74 (0.17)
In education	22, 0.54 (0.17)	22, 0.63 (0.20)	23, 0.62 (0.24)
Non-working	57, 0.74 (0.13)	56, 0.69 (0.19)	60, 0.72 (0.17)
Missing ^2^	1, 0.42 (-)	1, 0.63 (-)	1, 0.50 (-)
Household composition Other adults in the household	*H*(2) = 6.63 *	*H*(2) = 0.28	*H*(2) = 0.35
No	29, 0.74 (0.16)	33, 0.64 (0.21)	33, 0.69 (0.22)
Yes	149, 0.66 (0.19)	152, 0.62 (0.19)	162, 0.68 (0.19)
Missing	13, 0.63 (0.12)	15, 0.61 (0.21)	15, 0.70 (0.19)
Children in the household	*H*(2) = 10.41 **	*H*(2) = 2.65	*H*(2) = 2.98
No	119, 0.70 (0.17)	128, 0.64 (0.20)	134, 0.70 (0.21)
Yes	42, 0.61 (0.21)	38, 0.59 (0.19)	43, 0.66 (0.17)
Missing	30, 0.62 (0.17)	34, 0.60 (0.21)	33, 0.68 (0.17)
Monthly household net-income	*H*(5) = 11.04	*H*(5) = 3.86	*H*(5) = 5.27
≤EUR 450	5, 0.77 (0.13)	5, 0.71 (0.25)	4, 0.72 (0.20)
EUR 450–<1500	37, 0.68 (0.17)	39, 0.62 (0.18)	40, 0.71 (0.20)
EUR 1500–<2500	48, 0.72 (0.17)	57, 0.65 (0.20)	56, 0.71 (0.18)
EUR 2500–<4000	61, 0.66 (0.17)	60, 0.61 (0.21)	67, 0.68 (0.21)
≥EUR 4000	36, 0.59 (0.20)	35, 0.61 (0.18)	40, 0.64 (0.18)
Missing	4, 0.65 (0.32)	4, 0.55 (0.23)	3, 0.70 (0.20)

^1^ For the test statistics, *r* (Spearman’s rank correlation coefficient), *U* (Mann–Whitney U test), or *H* (Kruskal–Wallis test) values are reported, as the normality assumption for the situational stability index was not met. ^2^ *SD* could not be calculated because only one participant was assigned to “Missing”. * indicates *p* < 0.05. ** indicates *p* < 0.01. *** indicates *p* < 0.001.

## Data Availability

Data will be made available upon request.

## Supplementary Materials

The following supporting information can be downloaded at: https://www.mdpi.com/article/10.3390/nu15183967/s1, Table S1: Socio-demographic characteristics of completers and non-completers plus drop out analysis. Table S2: Description of the clusters for breakfast. Table S3: Description of the clusters for lunch. Table S4: Description of the clusters for dinner.
