# Supplementary material for: In Which Situations Do We Eat? A Diary Study on Eating Situations and Situational Stability
Source: Nutrients. 2023 Sep 14;15(18):3967. doi: 10.3390/nu15183967 (PMC10537183; doi:10.3390/nu15183967)
Supplement: Supplementary file 1 [file nutrients-15-03967-s001.zip › nutrients-2557303-supplementary.pdf]

Supplementary Material

Table S1. Socio-demographic characteristics of completers and non-completers plus drop-out analysis.

Socio-demographic characteristics	Completers (*N* = 230)	Non-completers (*N* = 251)	Logistic regression DV [1] = completion of diary study	
	**M(SD) / N (%)**	**M(SD) / N (%)**	**OR [2]**	**CI [3]**
Age	42.70 (17.25%)	38.04 (16.68%)	1.00	0.98 – 1.01
Sex				
Male	135 (58.7%)	133 (52.99)	Reference	
Female	95 (41.30%)	118 (47.01)	1.34	0.90 – 1.99
Employment status				
Full-time	102 (44.35%)	125 (49.80)	Reference	
Part-time	36 (15.65%)	28 (11.16)	1.99*	1.09 – 3.68
In education	24 (10.43%)	50 (19.92)	0.67	0.35 – 1.24
Non-working	67 (29.13%)	47 (18.73)	2.05*	1.15 – 3.71
Missing	1 (0.43%)	1 (0.40)	1.32	0.05 – 37.06
Household composition				
Other adults in the household				
No	37 (16.09%)	49 (19.52%)	Reference	
Yes	177 (76.96%)	184 (73.31%)	1.51	0.89 – 2.58
Missing	16 (6.96%)	18 (7.17%)	2.09	0.81 – 5.43
Children in the household				
No	147 (63.91%)	131 (52.19%)	Reference	
Yes	47 (20.43%)	68 (27.09%)	0.57*	0.35 – 0.94
Missing	36 (15.65%)	52 (20.72%)	0.56	0.31 – 1.00
Monthly household net-income				
≤EUR 450	5 (2.17%)	6 (2.39%)	0.91	0.23 – 3.45
EUR 450–<1500	42 (18.26%)	54 (21.51%)	0.82	0.46 – 1.44
EUR 1500–<2500	64 (27.83%)	68 (27.09%)	Reference	
EUR 2500–<4000	71 (30.87%)	75 (29.88%)	1.13	0.69 – 1.86
≥EUR 4000	44 (19.13%)	41 (16.33%)	1.42	0.79 – 2.58
Missing	4 (1.74%)	7 (2.79%)	0.76	0.18 – 2.84

[1] DV = dependent variable. [2] OR = odds ratios. [3] CI = confidence intervals. * indicates $p < .05$.

Our analysis indicated differences in drop-out rates for the socio-demographic characteristics of employment and children in the household: there was a higher drop-out rate among participants who worked full-time compared to participants who worked part-time or not at all. Possible explanations could be that financial incentives were less important for full-time employees, while simultaneously less time was available due to employment. Further, there was a higher drop-out rate among participants with children living in the household compared to participants without children in the household. Reasons for this might include having less available time to complete the survey or difficulties meeting the survey deadlines. Future research should increase incentives or facilitate participation for these subgroups.

Table S2. Description of the clusters for breakfast.

Cl [1]	$n_{breakfasts}$ [2]	$n_{participants}$ [3]	Situational dimensions				
			Location Mode (%)	Hunger Mode (%)	Social Mode (%)	Activity Mode (%)	Affect [4] Mode (%)
1	258	104	Home (100)	Satiated (100)	Alone (100)	With activity (55)	Low NA (52)
2	191	82	Home (100)	Hungry (100)	Alone (100)	With activity (58)	High NA (54)
3	133	57	Home (100)	Satiated (58)	With others (100)	Without activity (63)	Low NA (100)
4	104	48	Home (100)	Hungry (53)	With others (100)	With activity (55)	High NA (100)
5	50	24	Out of home (100)	Hungry (50)	Alone (78)	With activity (88)	High NA (74)
6	34	15	Out of home (100)	Hungry (65)	With others (76)	Without activity (97)	Low NA (76)

[1] Cl = Cluster.
[2] $n_{breakfasts}$ = Number of breakfasts per cluster. The total number of breakfasts was $N_{breakfast} = 770$.
[3] $n_{participants}$ = Number of participants who ate at least one breakfast in the respective cluster. In the analysis, we only included participants who ate at least one breakfast during the observed period ($N_{participants} = 191$).
[4] NA = Negative affect.

Table S3. Description of the clusters for lunch.

Cl [1]	$n_{lunches}$ [2]	$n_{participants}$ [3]	Situational dimensions				
			Location Mode (%)	Hunger Mode (%)	Social Mode (%)	Activity Mode (%)	Affect [4] Mode (%)
1	200	77	Home (100)	Satiated (56)	With others (56)	Without activity (100)	Low NA (100)
2	157	75	Home (100)	Satiated (57)	Alone (100)	With activity (59)	High NA (100)
3	139	66	Home (100)	Satiated (50)	Alone (64)	With activity (100)	Low NA (100)
4	103	52	Home (100)	Satiated (62)	With others (100)	Without activity (56)	High NA (100)
5	77	37	Out of home (100)	Hungry (100)	Alone (51)	Without activity (100)	High NA (55)
6	50	31	Out of home (100)	Satiated (100)	With others (56)	Without activity (100)	Low NA (62)
7	43	30	Out of home (100)	Satiated (53)	With others (51)	With activity (100)	High NA (100)
8	25	16	Out of home (100)	Hungry (56)	Alone (52)	With activity (100)	Low NA (100)

[1] Cl = Cluster.
[2] $n_{lunches}$ = Number of lunches per cluster. The total number of lunches was $N_{lunches} = 794$.
[3] $n_{participants}$ = Number of participants who ate at least one lunch in the respective cluster. In the analysis, we only included participants who ate at least one lunch during the observed period ($N_{participants} = 200$).
[4] NA = Negative affect.

Table S4. Description of the clusters for dinner.

Cl [1]	$n_{dinners}$ [2]	$n_{participants}$ [3]	Situational dimensions				
			Location Mode (%)	Hunger Mode (%)	Social Mode (%)	Activity Mode (%)	Affect [4] Mode (%)
1	268	92	Home (100)	Hungry (50)	With others (100)	Without activity (52)	Low NA (100)
2	199	71	Home (100)	Satiated (53)	Alone (100)	With activity (68)	Low NA (100)
3	192	91	Home (100)	Hungry (52)	Alone (51)	With activity (100)	High NA (100)
4	171	67	Home (100)	Satiated (56)	Alone (51)	Without activity (100)	High NA (100)
5	34	22	Out of home (100)	Hungry (53)	With others (62)	With activity (100)	High NA (79)
6	22	17	Out of home (100)	Hungry (73)	With others (100)	Without activity (100)	Low NA (64)
7	11	7	Out of home (100)	Hungry (55)	Alone (100)	Without activity (91)	Low NA (73)

[1] Cl = Cluster.

[2] $n_{dinners}$ = Number of dinners per cluster. The total number of dinners was $N_{dinners}$ = 897.

[3] $n_{participants}$ = Number of participants who ate at least one dinner in the respective cluster. In the analysis, we only included participants who ate at least one dinner during the observed period ($N_{participants}$ = 210).

[4] NA = Negative affect.
